# Three-Dimensional Reconstruction, by TEM Tomography, of the Ultrastructural Modifications Occurring in *Cucumis sativus* L. Mitochondria under Fe Deficiency

**DOI:** 10.1371/journal.pone.0129141

**Published:** 2015-06-24

**Authors:** Gianpiero Vigani, Franco Faoro, Anna Maria Ferretti, Francesca Cantele, Dario Maffi, Marcello Marelli, Mauro Maver, Irene Murgia, Graziano Zocchi

**Affiliations:** 1 Dipartimento di Scienze Agrarie e Ambientali—Produzione, Territorio, Agroenergia, Università degli Studi di Milano, Milano, Italy; 2 Istituto di Scienze e Tecnologie Molecolari, Consiglio Nazionale delle Ricerche, Milano, Italy; 3 Dipartimento di Chimica, Università degli Studi di Milano, Milano, Italy; 4 Dipartimento di Bioscienze, Università degli Studi di Milano, Milano, Italy; Institute for Sustainable Plant Protection, C.N.R., ITALY

## Abstract

**Background:**

Mitochondria, as recently suggested, might be involved in iron sensing and signalling pathways in plant cells. For a better understanding of the role of these organelles in mediating the Fe deficiency responses in plant cells, it is crucial to provide a full overview of their modifications occurring under Fe-limited conditions. The aim of this work is to characterize the ultrastructural as well as the biochemical changes occurring in leaf mitochondria of cucumber (*Cucumis sativus* L.) plants grown under Fe deficiency.

**Methodology/Results:**

Mitochondrial ultrastructure was investigated by transmission electron microscopy (TEM) and electron tomography techniques, which allowed a three-dimensional (3D) reconstruction of cellular structures. These analyses reveal that mitochondria isolated from cucumber leaves appear in the *cristae junction model* conformation and that Fe deficiency strongly alters both the number and the volume of cristae. The ultrastructural changes observed in mitochondria isolated from Fe-deficient leaves reflect a metabolic status characterized by a respiratory chain operating at a lower rate (orthodox-like conformation) with respect to mitochondria from control leaves.

**Conclusions:**

To our knowledge, this is the first report showing a 3D reconstruction of plant mitochondria. Furthermore, these results suggest that a detailed characterization of the link between changes in the ultrastructure and functionality of mitochondria during different nutritional conditions, can provide a successful approach to understand the role of these organelles in the plant response to Fe deficiency.

## Introduction

Iron (Fe) is an essential element for key metabolic reactions in plants, including the respiratory and photosynthetic electron transport chains located in the endosymbiotic organelles [1]. Fe deficiency represents a widespread agricultural problem in calcareous soils characterized by low Fe solubility [2, 3]. Iron deficiency compromises plant growth, thus reducing both crop yield and quality parameters. In humans, Fe deficiency is the most common micronutrient deficiency in the diet; Fe deficiency affects an estimated 2 billion people [4] with severe consequences for human health, especially in the developing countries [5].

Staple plants such as cereals and legumes represent an important source of Fe for human diet, and therefore a more complete understanding of plant Fe homeostasis, trafficking and transport to various plant organs is of high priority for plant physiologists. Indeed, research efforts can provide a platform-knowledge for improving and/or adopting new biofortification strategies in order to produce Fe-dense crops [5, 6]. Plants initiate various metabolic changes in response to Fe deficiency aimed at increasing Fe acquisition capacity. Such metabolic changes are strictly dependent on functional alterations occurring in essential cell compartments such as the mitochondria and chloroplasts [1, 7]. In turn, changes in the metabolic status of these organelles, such as those observed in response to an altered Fe nutritional status, exert profound effects on the whole plant cell, involving massive modifications in the transcript profiles of nuclear genes [1]. While mature chloroplasts are present only in green tissues, mitochondria are present everywhere in plant tissues. Mitochondria might be involved in regulation of Fe deficiency-induced responses by a retrograde signalling pathway [1]. Such organelles represent the powerhouse of the cell and are able to activate adaptation mechanisms in responses to abiotic stress ([8] and references therein). In plant cells, mitochondria share such roles with chloroplasts, thus creating specific cross-talks that help plant cells to adapt to the environmental changes.

Detailed insights about the regulation of mitochondrial processes are necessary to provide useful information for breeding programs addressed to maximize respiration and to minimize respiratory losses in harsh environments, thus enhancing plant yields [8].

Studies showing the functional characterization of the mitochondrial activity in Fe-deficient plants have been reported in the last decade ([9] and references therein). In general, low Fe availability affects respiratory chain through strong decreases in the complex I, II, III, IV and V activities. At the same time, the activities of alternative NAD(P)H dehydrogenases are strongly induced in Fe-deficient mitochondria, thus bypassing the loss of complex I and II activities [10–12]. However, very little information is present concerning the ultrastructural changes of mitochondria induced by Fe deficiency.

In the last decades, Transmission Electron Microscopy (TEM) analyses have helped to reveal basic cell structures in detail, allowing researchers to visualize their internal structure. However, TEM methodology provides only a two-dimensional (2D) view of thin cellular slices. Over the past fifteen years, Electron Tomography (ET) has emerged as a powerful tool to overcome the 2D limitation [13]; such technique yields three-dimensional (3D) structures by computationally combining a large number of 2D images of a given sample collected in a tomographic series. The resulting 3D models enable the investigation of the cellular compartments with unprecedented detail [13].

Three-dimensional characterization of mitochondria from animal cells have been object of deep investigation in the last decades [14]. However, the mitochondrial ultrastructure in plant cells has never been investigated at 3D level so far, and studies reporting the visualization of the real plant mitochondrial ultrastructure are still lacking. Electron tomography has been used in plant tissues to study thylakoid membranes [15] but no data on mitochondria have been reported so far. It should also be noted that ultrastructural changes induced in mitochondria by a low Fe availability are scarcely documented [16].

Here we show that Fe deficiency strongly affects mitochondrial function and ultrastructure in cucumber leaves. Particularly, we demonstrate that the cristae number and the intracristae surface area decreased in Fe-deficient leaf tissues and, for the first time, we report a 3D reconstruction of plant mitochondria obtained by electron tomography.

## Material and Methods

### Plant material and growth conditions

Seeds of cucumber (*Cucumis sativus* L. cv. Marketer) were surface-sterilized and sown in Agriperlite, watered with 0.1 mm CaSO_4_, allowed to germinate in the dark at 26°C for 3 d, and then 70 seedlings were transferred to a box containing 20 L of the following nutrient solution: 2 mM Ca(NO)_3_, 0.75 mM K_2_SO_4_, 0.65 mM MgSO_4_, 0.5 mM KH_2_PO_4_, 10 μM H_3_BO_3_, 1 μM MnSO_4_, 0.5 μM CuSO_4_, 0.5 μM ZnSO_4_, 0.05 μM (NH_4_)Mo_7_O_24_; 0.1 mM Fe(III)-EDTA was added for control plants but omitted in—Fe plants. The pH was adjusted to 6.0–6.2 with NaOH. Aerated hydroponic cultures were maintained in a growth chamber with a day:night regime of 16:8 h and a photosynthetic photon flux density (PPFD) of 200 μmol photons m^−2^ s^−1^. The temperature was 18°C in the dark and 24°C in the light.

### Chlorophyll quantification, oxygen evolution and consumption

For each measurement, segments of expanded 10 d old *Cucumis sativus* leaves (around 2cm x 2cm) was cut, weighed and oxygen evolution measured, by using the Clark-type oxygen electrode (Hansatech Ltd, King's Lynn, Norfolk), as described in [17], at 200 μE m^-2^ s ^-1^ illumination. Rates were analysed with Oxylab V1.15 software. Leaf segments were then removed from chamber and put in a vial containing 2–6 ml dimethylformamide for chlorophyll extraction and quantification, according to [18]. Leaf segments were excised under water at room temperature from plants illuminated for several hours. Leaf O_2_ consumption rates were measured as decrease in O_2_ concentration in an aqueous phase with a Clark-type O_2_ electrode (Hansatech) at 25°C. Calibration was made from the difference in signal between aerated water and Na-dithionite saturated water. Addition of 2 mM KCN was made directly to roots in the measurement chamber. Roots were preincubated with 2 mM salycilhydroxamic acid (SHAM) for 20 min before measurement, according to [10, 19]. Data collected are from three independent experiments

### Leaf physiological parameters

Gas exchange measurements were performed with a portable photosynthesis system (CIRAS-2, PP System, USA). Measurements were taken on young, fully expanded, intact leaves of 10-d old cucumber plants. Net CO_2_ assimilation rate, stomatal conductance and transpiration were assessed setting the CO_2_ concentration of the instrument at 400 μmol mol^-1^, 50% relative humidity, 28°C chamber temperature, 500 mL min^-1^ airflow and a photon flux density of 1500 μmol photons m^-2^ s^-1^. The instrument was stabilized according to manufacturer guidelines. Data collected are from three independent experiments

### Metal content determination

Sampled tissues were dried and then mineralized in HNO_3_ by using a Microwave Digestion System (Multiwave ECO). Metal content was determined by Inductively Coupled Plasma-Mass Spectrometry (ICP-MS, aurora M90 BRUKER). Data collected are from three independent experiments

### Purification of mitochondria and chloroplasts and western blot analysis

Mitochondria and chloroplasts were purified according to Rödiger et al. [20] from expanded leaves of 10 d old cucumber plant grown in the presence (C) and in the absence of Fe (-Fe) in the medium. The purified fractions were loaded on a discontinuous SDS-polyacrylamide gel (3.75% (w/v) acrylamide stacking gel and 10–15% (w/v) acrylamide separating gel). Electrophoretic transfer to nitrocellulose membrane filters (Sigma, Milan, Italy) was performed according to [10]. Five different antibodies were used: two monoclonals against maize alternative oxidase (AOX) and porin [10, 21], two polyclonals against the yeast Rieske and the cytochrome *c* [10, 22] and a polyclonal against *Arabidopsis* translocase of chloroplast envelope (Toc33) [20]. The incubation in primary antibody, diluted in block buffer, was carried out for 2 h at room temperature. After rinsing with TBS-TM (Tris-buffered saline, 0.1% Tween-20, 5% commercial dried skimmed milk) for polyclonal antibodies, or with PBS-TB (phosphate buffered saline, 0.1% Tween-20, 1% BSA) for monoclonal antibodies, the nitrocellulose membranes were incubated at room temperature for 2 h with a 1:10000 diluted secondary antibody (alkaline phosphatase-conjugated anti-rabbit for polyclonals or anti-mouse for monoclonals, Sigma). Filters were rinsed in TBS-T (Tris-buffered saline, 0.1% Tween-20) or PBS-T (phosphate-buffered saline, 0.1% Tween-20) and incubated in 5-bromo-4-chloro 3-indolyl phosphate and nitroblue tetrazolium (FAST BCIP/NBT, Sigma) for signal detection.

### Transmission electron microscopy

Samples of Fe-deficient and control leaf tissues were fixed in a mixture of 3% (v/v) glutaraldehyde and 2% (w/v) paraformaldehyde in 0.1 mM phosphate buffer, pH 7, overnight at room temperature. Samples were subsequently post fixed with 1% (w/v) osmium tetroxide in the same buffer for 1 h at 4°C and dehydrated in a graded ethanol series before being embedded in SPURR resin (Electron Microscopy Sciences, Washington, PA, USA) according to [10]. Ultrathin sections were cut from at least three leaf samples from different plants and contrasted with uranyl acetate and lead citrate and examined with a Jeol JEM-100 SX TEM at 80 kV and with Zeiss LIBRA 200FE-HR TEM (transmission electron microscope), operating at 200 kV and equipped with an in-column omega filter for energy selective imaging. We collected more than one hundred images for treatments obtained from Fe-deficient and control plants of three independent experiments. By using Adobe Photoshop tools, the number of mitochondrial cristae as well as the relative intracristae surface area (total intracristae surface area/ mitochondrion area, expressed as % ratio) were determined on hundred different mitochondria for both treatments taken from all the collected TEM images.

### Electron tomography

Leaf tissues of both Fe-deficient and control plants were fixed as reported for the TEM analysis and sections of about 150 nm thickness were obtained.

The tomography acquisitions were performed using Zeiss LIBRA 200FE-HR TEM, operating at 200 kV and equipped with an in-column omega filter for energy selective imaging and diffraction. The tomographic series were collected with a Fischione 2040 Dual-Axis Tomography Holder, following the dual-axis strategy [23].

A total of 122 images were acquired, consisting of two tilt-series of 61 images each, collected with a tilt angle step of 2 degrees with a range from -60 to +60 degrees for α angle, then the second series were acquired after a grid rotation of γ 90 degrees in plane. Prior to the tomography acquisition, gold nanoparticles (spherical NPs having a mean diameter of 25.7 +/- 4.0 nm estimated by means PEBBLES and PEBBLESJUGGLER [24]) were deposited onto both side of the thin section as fiducial markers. The two series were combined together in order to obtain complete information on the 3D structure of the feature analysed. The gold particles positions were tracked for each series using the program IMOD [25] and their coordinates were exported in a text file to be processed. The quality of the alignment can be evaluated visually in the reconstructed tomograms. The magnification chosen is 16000x, the image resolution 0.94 nm/pixel. The tomography of the most representative C and—Fe mitochondria were reported.

The 3D reconstruction is obtained by using of weighted back-projection algorithm and simultaneous alignment method followed by local refinement [26]. The final tomogram was segmented by use of the program JUST [27] and the interesting objects evidenced. The tracings from all sections are modelled as 3D surfaces and displayed as a 3D model by the program Avizo (FEI, SAS).

## Results

### Fe deficiency affects several physiological parameters in cucumber leaves

**Fig 1:**
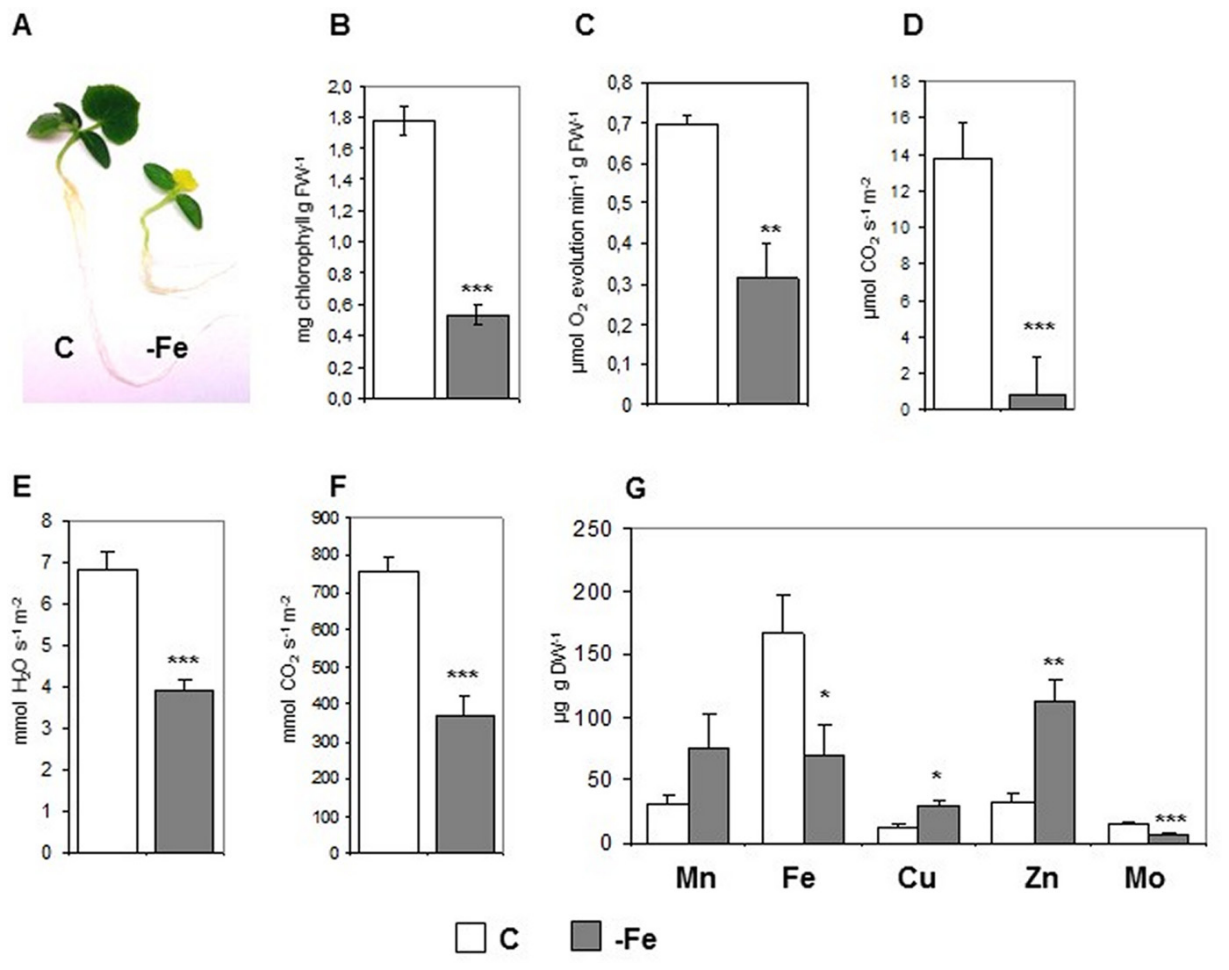
Physiological characterization of leaves from cucumber plants grown in control condition (C) or under Fe deficiency (–Fe). **(A)** Phenotype of 10 days old cucumber plants grown under control condition (C) or under Fe deficiency (-Fe). The following parameters have been evaluated, on expanded leaves of plants grown as in (A): **(B)** total chlorophyll concentration, expressed as mg chlorophyll g FW^-1^; **(C)** O_2_ evolution, expressed as μmol O_2_ min^-1^ g FW^-1^; **(D)** net photosynthesis expressed as μmol CO_2_ consumed s^-1^ m^-2^; **(E)** evapotranspiration, expressed as mmol H_2_O s^-1^m^-2^; **(F)** stomatal conductance, expressed as mmol CO_2_ s^-1^m^-2^; **(G)** manganese (Mn), iron (Fe), copper (Cu), zinc (Zn), molybdenum (Mo) concentrations, expressed as μg g DW^-1^. Data are means ± SE of at least three independent experiments. Student t-test was used to analyse statistical significance with respect to controls. *:p<0,05; **:p<0,01; ***:p<0,001.

Ten-day old cucumber plants grown under Fe deficiency (-Fe) displayed yellowish leaves and a reduced growth when compared to control (C) plants (Fig 1A). The photosynthetic process is strongly affected in leaves from plants grown under Fe deficiency: indeed, a reduction in total chlorophyll concentration (Fig 1B) O_2_ evolution (Fig 1C), CO_2_ consumption (Fig 1D), transpiration (Fig 1E) and stomatal conductance (Fig 1F), was observed.

The quantification of metal content (metallome) in the above described leaf tissues showed, as expected, a drastic drop of Fe content under Fe deficiency, together with a decrease in molybdenum (Mo) content (Fig 1G). On the contrary, an increase in other divalent metals, such as copper (Cu) and zinc (Zn) occurred under Fe deficiency (Fig 1G).

### Fe deficiency affects mitochondrial function in cucumber leaves

**Fig 2:**
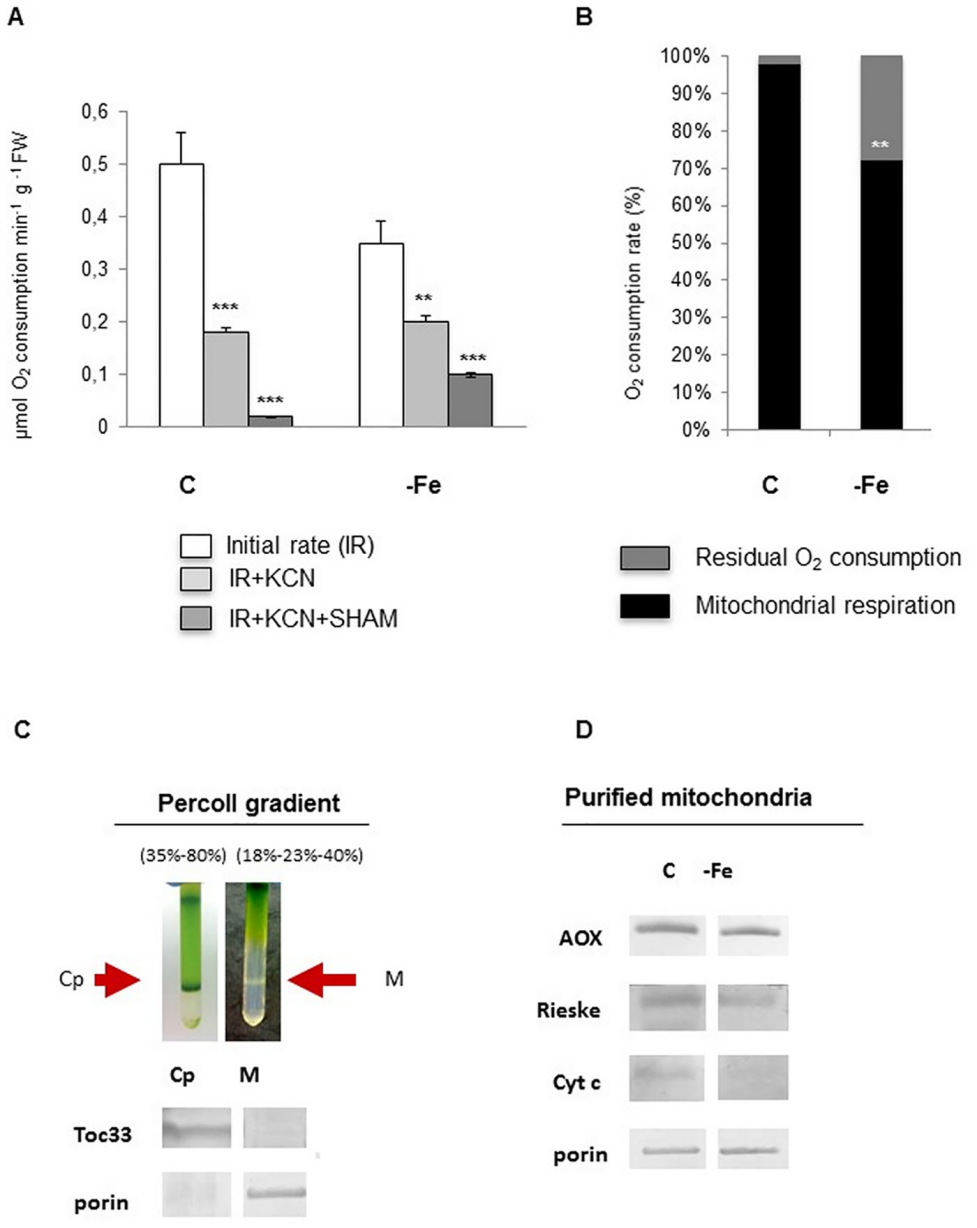
Mitochondrial functionality of leaves from cucumber plants grown in control conditions (C) or under Fe deficiency (-Fe). **(A)** *In vivo* O_2_ consumption rates expressed as μmol O_2_ consumed min^-1^ g FW^-1^. The total O_2_ consumption rate (IR), as well as the residual O_2_ consumption, measured by using KCN (inhibiting cytochrome c oxidase) (IR + KCN) or KCN in combination with SHAM (inhibiting alternative oxidase) (IR + KCN +SHAM) were measured. Values represent mean ±SE of three independent measurements. **(B)** For each growth condition, the residual O_2_ consumption (IR +KCN+SHAM), reported in (A), is shown as % value, with respect to correspondent IR. Mitochondrial respiration is the difference between IR and residual O_2_ consumption. Student t-test was used to analyse statistical significance with respect to controls. *:p<0,05; **:p<0,01; ***:p<0,001. **(C)** Mitochondria (M) and chloroplasts (Cp) were purified from control plants by percoll gradient; the Cp and M fractions at 35/80% percoll and 23/40% percoll layers interfaces, respectively, are indicated with arrows in the gradient tubes. The purified Cp and M fractions were tested by western blot, by using antibodies against two protein markers: Toc33 and porin, specific for Cp, and M, respectively. 10 μg total proteins were loaded in each lane. **(D)** Western blot analysis of three different mitochondrial Fe-containing proteins (AOX, Rieske, cyt c) on mitochondria purified from leaves of control (C) or Fe-deficient (-Fe) plants. Porin was used as loading control; 10 μg total proteins were loaded in each lane. The results are representative of tree independent experiments.

The rate of O_2_ consumption in leaf tissues from Fe-deficient cucumber plants was investigated, along with the non-respiratory O_2_ consumption, by using specific inhibitors of the O_2_-consuming terminals of the respiratory chain (salicylhydroxamic acid, SHAM, which inhibits the alternative oxidase, and KCN, which inhibits the cytochrome c oxidase). A decrease in the mitochondrial respiratory activity was observed under Fe deficiency, when comparing initial rates (IR) in control and Fe-deficient leaves (Fig 2A). Moreover, similarly to what already observed in Fe-deficient cucumber roots [10], Fe-deficient leaves showed a lower residual consumption of O_2_ than control after addition of inhibitors of the respiratory activity (KCN+SHAM), suggesting that other O_2_-consuming reactions are activated under Fe deficiency. However, the O_2_ consumption rate attributable to mitochondrial respiration (IR minus residual O_2_ consumption) decreased in-Fe leaf when compared with control conditions (Fig 2A and 2B).

Mitochondria were isolated from leaves of plants grown in both conditions of Fe supply; the purity of mitochondrial fractions from chloroplast contamination was tested by western blot analysis, by using an antibody against Toc33 (as a chloroplast marker), and an antibody against porin (as a mitochondrial marker) (Fig 2C). As a control, chloroplasts were also purified and tested with the same antibodies (Fig 2C).

A decrease in the expression level of three Fe-containing proteins of the respiratory chain, i.e. the alternative oxidase (AOX), Rieske protein and the cytochrome *c* (cyt *c*), was observed in purified mitochondria from—Fe leaves when compared to those purified from control leaves (Fig 2D), in accordance with the reduction in respiratory activity observed under Fe deficiency (Fig 2B).

### Fe deficiency affects the mitochondrial ultrastructure of cucumber leaves as revealed by Transmission Electron Microscopy

**Fig 3:**
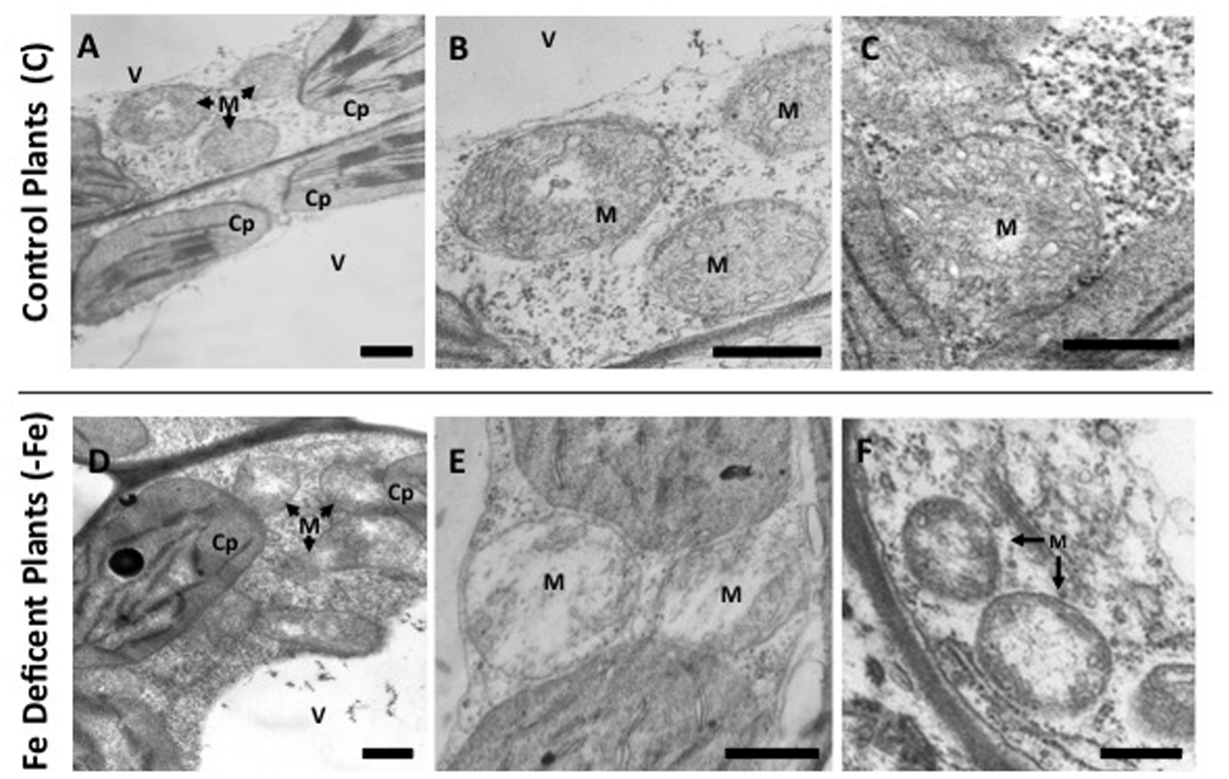
Transmission electron microscopy (TEM) analysis of leaf tissues from cucumber plants grown in control condition (C) or under Fe deficiency (-Fe). Control leaf tissues (**A-C**) and -Fe leaf tissues (**D-F**). All scale bars = 500 nm. M: mitochondria; C: chloroplast; V: vacuole.

Ultrathin sections obtained from mesophyll of cucumber plant leaves grown under control (C) or under Fe deficiency (-Fe) were observed by TEM at different magnifications (Fig 3). Although the most evident morphological alteration in Fe-deficient leaf tissues was related to chloroplasts, which appeared swollen, and with deranged thylakoids (Fig 3A), Fe deficiency also affected mitochondria ultrastructure. Mitochondria from control leaves displayed homogenous matrices as well as regular internal cristae (Fig 3A, 3B and 3C). Instead, under Fe deficiency, a heterogeneous morphology of the shape of mitochondria was observed: few of them appeared only slightly altered, but the majority of them displayed a lower matrix density and a reduced number of cristae when compared to control plants (Fig 3D, 3E and 3F).

The above described ultrastructural alterations were observed in all the examined leaf samples from three independent experiments. We processed more than one hundred TEM images for each sample and we determined the number of cristae as well as the relative intracristae surface area (intracristae surface area *per* mitochondrion) on one hundred mitochondria randomly selected for each sample (Table 1). Under Fe deficiency, the number of cristae per mitochondrion decreased by about 56% (p< 0,001), while the relative intracristae surface area decreased by about 46% (p< 0,001) (Table 1).

**Table 1:** Determination of the cristae number *per* mitochondrion and the relative intracristae surface area (intracristae surface area *per* mitochondrion surface area, expressed as % ratio).

	C	-Fe
Number of cristae /mitochondrion	25.08 ± 10.61	10.95 ± 5.76 ***
Relative intracristae surface area (%)	14.01 ± 9.14	7.52 ± 3.94 ***

Mean values ± SD are from analysis of one hundred mitochondria randomly selected from inclusions obtained from three independent biological samples. Student t-test was used to analyse statistical significance with respect to controls.

***:p<0,001

### Fe deficiency affects the mitochondrial ultrastructure of cucumber leaves as revealed by Electron Tomography

**Fig 4:**
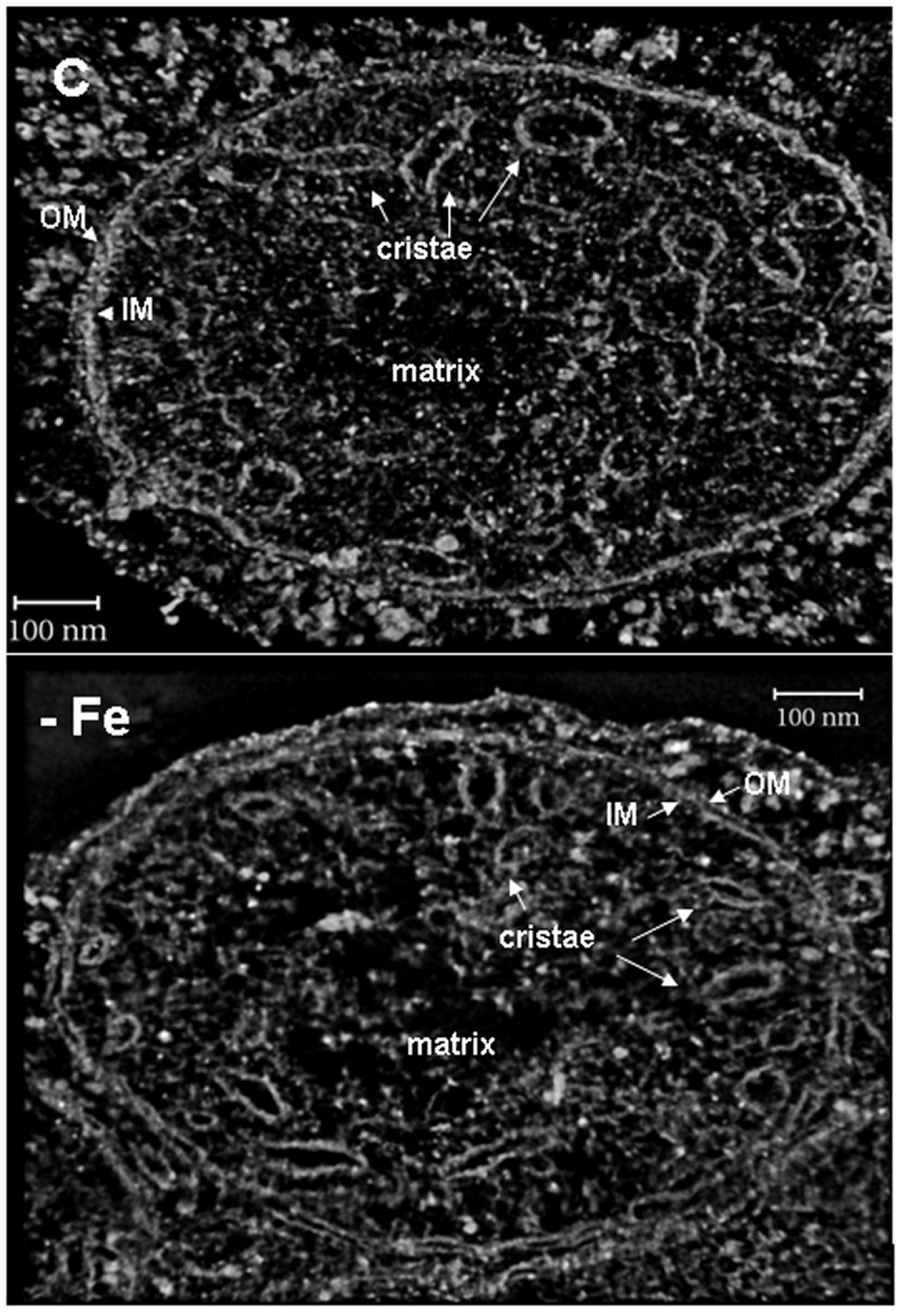
Tomograms of leaf mitochondria from cucumber plants grown in control condition (C) or under Fe deficiency (-Fe). Volume rendering view of two mitochondria tomograms, from control (C) or Fe deficient (-Fe) plant. Cristae, outer (OM) and inner (IM) membranes are clearly recognised. The pictures show the reduced amount of cristae in the-Fe mitochondria, especially in the central area.

The most representative mitochondria showing an altered morphology under Fe deficiency were selected for tomographic reconstructions with the dual-axis strategy [14, 23, 26]. A total number of seven maps were reconstructed. In Fig 4 examples of the results in C and -Fe mitochondria are reported.

**Fig 5:**
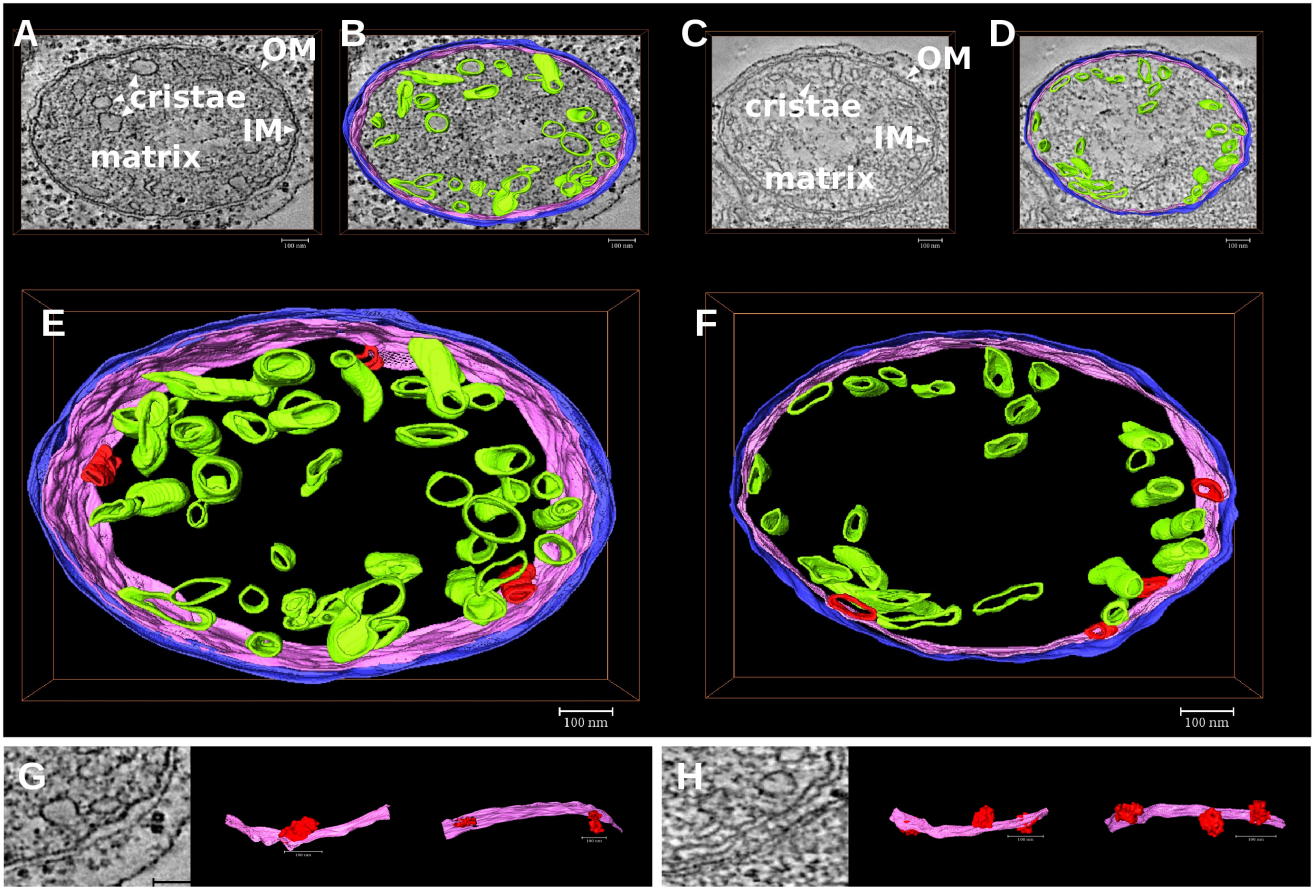
Three-dimensional models of leaf mitochondria from cucumber plants grown in control condition (C) or under Fe deficiency (-Fe). Different colours were used for the rendering of the different suborganellar structures: magenta for inner membranes (IM), blue for outer membranes (OM), green for cristae and red for cristae junctions. **(A-D)** Tomographic slices of mitochondria from control (A,B) and Fe-deficient (C,D) plants; the inner membrane (IM), the outer membrane (OM), and the matrix enclosed by the IM are indicated; in particular, the rendering of the mitochondria structures from control (B) and Fe-deficient (D) plants, superimposed on the tomographic slices, is reported. **(E,F)** Three-dimensional models of the mitochondria from control (E) or Fe-deficient (F) plants. **(G,H)** Details of mitochondrial cristae junctions, in red, identified in mitochondrial from control (G) or Fe-deficient (H) plants. Scale bars 100 nm.

In order to create a three-dimensional model, the tomograms were segmented by tracing different suborganellar components: the outer membrane (OM), the inner boundary membrane (IM), the cristae and the cristae junctions (Fig 5). Mitochondria displayed the so-called *cristae junction model*, were cristae are present as separate vesicles in the matrix; six discrete mitochondrial compartments could be recognized on a structural basis: outer membrane (OM), intermembrane space (IMS), inner boundary membrane (IM), cristae membrane, intracristae space, and matrix (Fig 5). Accordingly to TEM results reported in Fig 3, the 3D model of mitochondria from Fe-deficient leaves (Fig 5F and S2 Movie) displayed a lower cristae number as well as a decreased intracristae space when compared to the 3D model of mitochondria from control leaves (Fig 5E and S1 Movie). Additionally, we identified some cristae linked to the inner membrane as reported in Fig 5G and 5H.

## Discussion

Iron deficiency strongly affects mitochondrial function in plants. As Fe is required in large amount for the mitochondrial electron transport chain activity, insufficient Fe availability decreases the activity of the respiratory chain and mitochondria undergo a general metabolic reprogramming [11,12]. Such functional alterations might reflect changes in the mitochondrial ultrastructure. Indeed several observations indicate that the production of mitochondrial energy may be controlled by structural rearrangements of the organelle such as the remodelling of cristae morphology.

So far, few reports concerning the ultrastructural modification of mitochondria under Fe deficiency are available and a detailed characterization is lacking [10, 28]. Here, we performed a 3D reconstruction of mitochondria by electron tomography, combining a costless material preparation with an effective alignment method of tomographic series. Indeed tomograms of chemically-fixed and plastic-embedded material from cucumber leaves were collected as dual-axis tilt series and then aligned by using the simultaneous alignment method that greatly improve the quality of tomograms when compared with single-axis methods [23, 26].

Here we showed that the 3D models obtained from mitochondria, are similar to the so called *cristae junction model* [29]. Three different models of mitochondrial inner membrane topology have been observed so far, mainly in animal cells. In the first model, named *baffle model*, the IM is convoluted in a baffle-like manner with broad openings towards the intercristae space [30]. The second, named *Septa model*, is characterised by sheets of IM which are spanned like septa trough the matrix separating it into several distinct compartments [31]. The third, named *cristae junction model*, is characterized by cristae connected to the inner boundary membrane via tubular structures [32]. These structures, termed cristae junctions were discovered by EM tomography leading to the establishment of this as a currently accepted model of mitochondrial IM ([33] and references therein). Therefore, six discrete mitochondrial compartments can be recognized on a structural basis: outer membrane, intermembrane space, inner boundary membrane, cristae membrane, intracristae space and matrix. By analysing one hundred different mitochondria for each treatments, we observed a significant decrease of both the number of cristae and the relative intracristae surface area in Fe-deficient leaf mitochondria. This analysis revealed a great variability of the considered parameters in both treatments, suggesting that mitochondrial ultrastructure is highly dynamic. However, the large number of images processed allowed us to establish that the observed differences are statistically significant.

Such observed alterations might reflect a particular metabolic status of mitochondria; indeed, the morphology of the inner mitochondrial membrane changes with the metabolic state of the mitochondria [34–36]. Two mitochondrial conformations, reflecting a specific organelle status, have been observed so far: one defined as *orthodox*, corresponding to an expanded matrix volume and a compressed intracristae space and a second one defined as a *condensed* conformation, corresponding to a partial matrix contraction, with dilated intracristae spaces [14]. Mitochondria display a condensed conformation during maximum respiratory rate and in the presence of excess ADP and other respiratory substrate (state 3 of respiration process), but revert to an orthodox morphology after entering the state 4 of respiration, characterized by a reduced respiration due to the depletion of ADP [29, 34]. Furthermore, the switch from an *orthodox* to a *condensed* conformation, during mitochondrial biogenesis in maize embryo, is indicative of the changing biochemistry of the organelle as it switches from being reliant on the provision of electrons from external NADH dehydrogenases to the newly assembled TCA cycle [37]. The switch between these two conformations is a dynamic process, which depends on the metabolic status of the cell.

We observed that under Fe deficiency the respiratory activity, determined as O_2_ consumption rate attributable to mitochondria, decreased with respect to the control: these results suggest that, under Fe deficiency, the decrease in the number of cristae as well as the contraction of intracristae space might induce a prevalent mitochondrial *orthodox*-like conformation in the condriome. Therefore, when Fe is scarcely available, a decrease in the number of respiratory units and, in turn, a reduction of cristae surface in mitochondria, would occur. Additionally, the contraction of the intracristae space would greatly concentrate protons thus providing a suitable chemiosmotic potential to maintain mitochondria at work.

Such series of events occurring when Fe availability is compromised, would explain the tight link between the alterations of mitochondrial functionality occurring under Fe deficiency, as described in our lab as well as by other research groups ([16] and references therein), and the ultrastructural changes of such organelles reported in the present work.

In conclusion, the electron tomography of mitochondria revealed the detailed structure of such organelle as well as the changes occurring under Fe deficiency. Both the number of cristae and the intracristae surface area of mitochondria decrease in Fe-deficient leaf. The results presented in this work reinforce the link between metabolic adjustment and remodelling of mitochondrial ultrastructure. The molecular mechanism of such remodelling in plant is still not well known, whereas recent evidence in animal cells demonstrate that the change in the cristae shape plays a crucial role in the assembly stability of respiratory supercomplexes and in the mitochondrial respiratory efficiency [38]. When Fe is scarcely available, mitochondria lose their role as the powerhouse of the cell, and the cell undergoes to complex metabolic reprogramming [16].

The tight link between metabolic reprogramming and structural remodelling, as discussed in the present work, supports the need for a deep investigation of the molecular mechanisms responsible for the mitochondrial membrane remodelling in Fe-deficient plants. Such investigation has the potential to identify important molecular players in the plasticity of membrane morphology and, in turn, in the functional efficiency of mitochondria under Fe deficiency.

## Supporting Information

S1 Movie 3D model of mitochondria from control plant. Different colours were used for the rendering of the different suborganellar structures: magenta for inner membranes (IM), blue for outer membranes (OM), green for cristae and red for cristae junctions.
(MPG)

S2 Movie 3D model of mitochondria from Fe-deficient plant. Different colours were used for the rendering of the different suborganellar structures: magenta for inner membranes (IM), blue for outer membranes (OM), green for cristae and red for cristae junctions.
(MPG)
